# How Fast Is Your Body Motion? Determining a Sufficient Frame Rate for an Optical Motion Tracking System Using Passive Markers

**DOI:** 10.1371/journal.pone.0150993

**Published:** 2016-03-11

**Authors:** Min-Ho Song, Rolf Inge Godøy

**Affiliations:** fourMs Group, Department of Musicology, University of Oslo, Oslo, Norway; KSAU-HS, SAUDI ARABIA

## Abstract

This paper addresses how to determine a sufficient frame (sampling) rate for an optical motion tracking system using passive reflective markers. When using passive markers for the optical motion tracking, avoiding identity confusion between the markers becomes a problem as the speed of motion increases, necessitating a higher frame rate to avoid a failure of the motion tracking caused by marker confusions and/or dropouts. Initially, one might believe that the Nyquist-Shannon sampling rate estimated from the assumed maximal temporal variation of a motion (i.e. a sampling rate at least twice that of the maximum motion frequency) could be the complete solution to the problem. However, this paper shows that also the spatial distance between the markers should be taken into account in determining the suitable frame rate of an optical motion tracking with passive markers. In this paper, a frame rate criterion for the optical tracking using passive markers is theoretically derived and also experimentally verified using a high-quality optical motion tracking system. Both the theoretical and the experimental results showed that the minimum frame rate is proportional to the ratio between the maximum speed of the motion and the minimum spacing between markers, and may also be predicted precisely if the proportional constant is known in advance. The inverse of the proportional constant is here defined as the *tracking efficiency constant* and it can be easily determined with some test measurements. Moreover, this newly defined constant can provide a new way of evaluating the tracking algorithm performance of an optical tracking system.

## Introduction

When studying human body motion, optical marker-based tracking technology is generally considered one of the best-suited motion data acquisition methods. The technology can record a motion with high spatial precision (a resolution of less than a millimeter) and high temporal resolution (upward to several hundred Hz) [1–3]. Moreover, it is known as the least obtrusive motion tracking method, which allows a human body to move at high degrees-of-freedom [4].

To acquire motion data using optical marker-based technology, reflective markers are attached to a human body and/or a target structure (e.g. a musical instrument or other handheld object). These markers are spatially and temporally sampled using multiple high-speed infrared (IR) cameras. The trajectories of the markers in the three-dimensional space along the time axis are calculated and further processing of these trajectories can provide us with important motion feature data such as velocity, acceleration, jerk, etc. These motion features are used in many areas such as clinical analysis for rehabilitation, sports analysis for athletics, virtual reality, and also for musical performance analysis (see e.g. [3, 5–9]).

Marker-based motion tracking can be performed using either passive markers or active markers. A measurement process using passive markers is rather convenient, only necessitating the attachment of independent retro-reflective markers to target’s skin or clothing, while active markers need supplementary equipment such as batteries and power cables for self-emitting elements (mostly LEDs with distinct emitting patterns) that are used for marker identification. However, the convenience of the passive markers in the measurement process generally causes subsequent challenges in the post processing of motion tracking data for rapid motions with high level of marker proximity. This is so because the similarity of the passive markers causes the system to confuse the identity of the markers when they are close to each other. Although most professional optical motion tracking systems are equipped with various automatic marker identifying algorithms [10–12] for applications to passive markers, in practice there is usually a high risk for a system to confuse the trajectory of one marker with that of another, quite often necessitating a rather extensive manual editing by a user [13] (or even fail to reconstruct the trajectories).

The ambiguity of passive markers comes from the fact that in most cases of motion capture recordings there is more than one data point to trace in sampled images along the time axis. As a general model of this problem, we can think of relevant research in fluid mechanics, where Malik et al. [14] defined a measure of tracking difficulty for the so-called Particle Tracking Velocimetry (PTV) technique, as *the ratio of the average particle (marker) spacing to the mean distance moved by particles during one imaging time step*. This ratio may serve as a predictor of the difficulty with identifying the trajectories of data points in close proximity at high speeds. To overcome the problem, the main strategy to avoid tracking ambiguity, from the early work in the 1970’s to more recent work (which is also tends to use current high-end equipment), is the “predict and confirm” strategy [13–16]. In the prediction stage, a search neighborhood (also called as *prediction error* [10] or *predictor radius* [11] or *prediction radius* [12]) for each marker in the next frame is predetermined using the anticipated motion data from the previous and current frames [15]. Restricting the search neighborhood, the number of searches for marker labels decreases, and successively incoming data from future frames remove remaining ambiguity, finally confirming the labeling of markers.

It is noteworthy that a minimal displacement condition is needed for the tracking strategy to work well in practical situations [13–14]. In other words, a time interval between two successive sampling frames should be short enough to avoid rapid changes in the marker trajectories. This may be a contradictory condition because the tracking ambiguity usually occurs when a motion undergoes fast or sudden acceleration. Although the assumption is not sufficiently discussed in relevant literature [13–14], we believe that the minimal displacement condition is the crucial factor in how to select a frame rate (number of frames per second) in order for an optical motion tracking system using passive markers to work well. To achieve a successful optical tracking with passive markers in all cases, the frame rate of the system should be high enough to satisfy the minimal displacement condition of the tracking algorithm.

The objective of this paper is to suggest guidelines for determining a sufficient frame rate for an optical motion tracking system using passive markers. During the last two decades, human motion tracking systems have become popular in many research contexts (see [17–18] for intensive reviews), yet it is surprising that, to our knowledge, no relevant reports can be found about this problem using passive markers. Some researches discuss various sources of error in human motion tracking systems (e.g. [19–21]), but seem to mainly focus on measurement noise, latency and sampling duration problems, not on the sample rate issue. Bishop et al. [19, p.74] shortly mentioned that choosing a sensor sample rate for general tracking methods should be in accordance with the famous Nyquist-Shannon criterion [22–23]. However, we believe that the Nyquist-Shannon Sampling Rate (NSSR) cannot alone be the basis for determining a sufficient frame rate for the passive marker use as presented in this paper. The ambiguity of marker trajectories is certainly related to the speed (temporal aspect) of a motion, but our claim here is that it is also related to the proximity (spatial aspect) of the markers. This NSSR is a necessary condition to satisfy for the temporal aspect, but the proximity of the markers is an additional constraint here, necessitating a higher frame rate than the NSSR. When applying marker-based optical motion tracking to more large-scale human body motion such as in gait or in various sports activities, this constraint of marker proximity may not be a critical issue. However, some small-scale and rapid motions such as pianists’ finger motion in musical performance [24–25]–which is the authors’ main research interest–definitely involves a high level of marker proximity. This marker proximity can in fact be one of the reason for that optical motion tracking systems using passive markers often end up with severely confused and truncated trajectories although the equipment has descent tracking algorithms and high frame rates, generally considered sufficient for full-body or other large-scale motion tracking.

In this paper, the minimum frame rate required for an optical motion measurement using passive markers is derived theoretically and its validity is tested in two simple experiments. The critical moment in the motion tracking is when there exists a tracking-obstructing marker nearby another marker, which is approaching the obstructer at maximum speed. Applying the minimum displacement condition to avoid potential marker confusion, a sufficient frame rate can be determined with three numbers—the maximum speed, the minimum distance (spacing) between the markers, and a proportional constant. We have here defined the inverse of the proportional constant as the “tracking efficiency constant”, a constant that can measure the efficiency of the optical motion tracking system. In the ensuing experiments using a professional optical motion tracking system, periodic motions were observed with different marker spacing setups. From the experiments, speed and spacing relation with respect to the minimum sampling rate were verified. Also, the tracking efficiency constant could be easily determined during several test observations and furthermore showed consistency in predicting the frame rate requirement for given speeds and marker setups.

## Theoretical Formulation

### Notations

**Fig 1:**
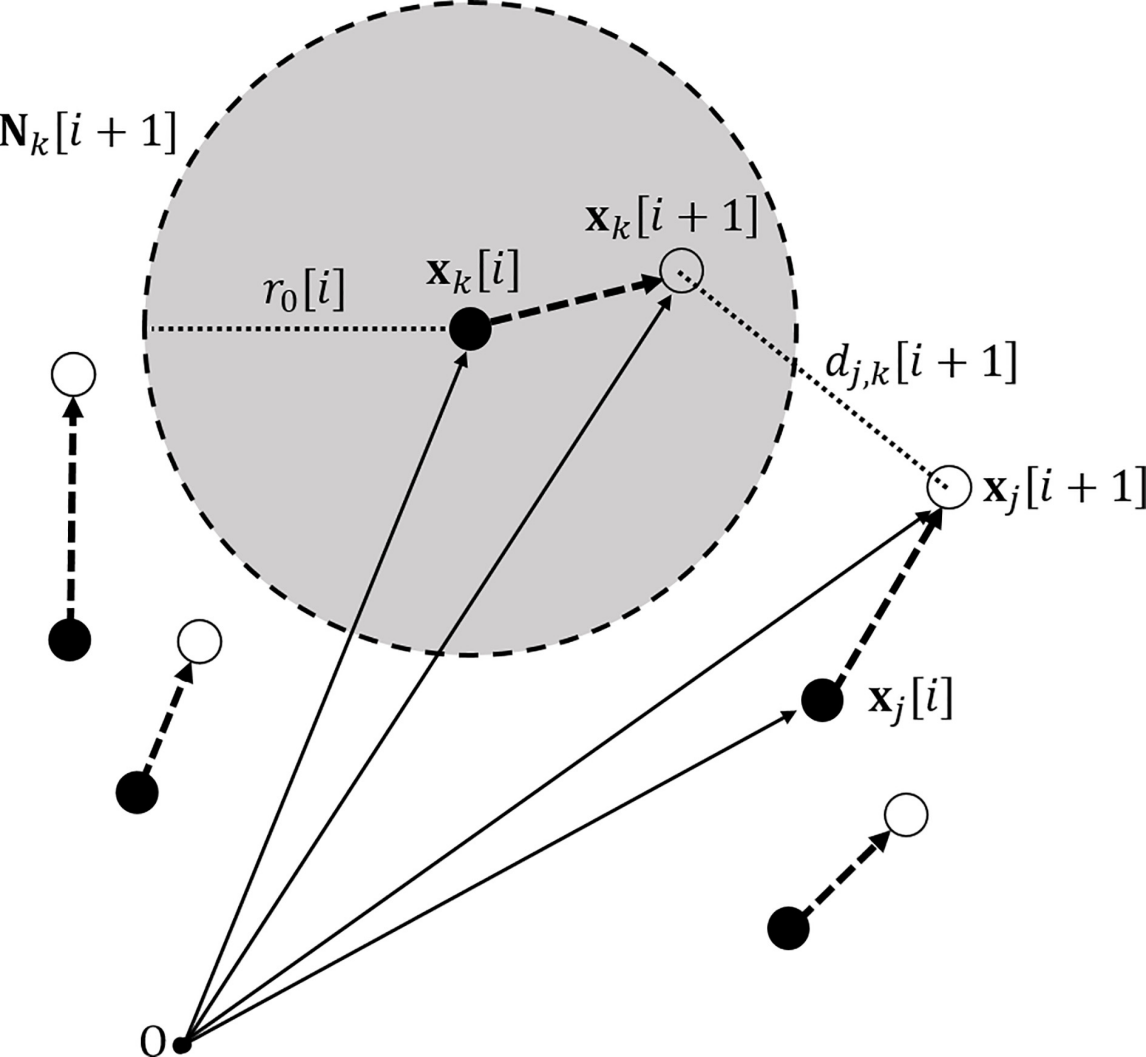
The illustration of multiple sampled points in the current frame and the next frame. The grey ball with the radius *r*_0_[*i*] denotes the search neighborhood of *k*-th marker for frame [*i* + 1] and the figure illustrates the case when the *k*-th marker in the next frame is within a given search neighborhood. The distance example *d*_*j*,*k*_[*i* + 1] is given for the two markers in frame [*i* + 1] where subscripts *j* and *k* denote the corresponding markers. The point O represents the coordinate origin for the camera system.

For simplicity, let the bracket [⋅] represents a frame index of the motion tracking data. Let us denote the position of *j*-th marker in frame [*i*] by the vector **x**_*j*_[*i*], where **x** is either 2-D or 3-D position vector and let *d*_*l*,*m*_[*i*] be the distance between two markers in frame [*i*] where subscripts *l* and *m* denote the corresponding markers (Fig 1). A unit time step between two successive frames is denoted with Δ*t*, which is the inverse of the frame rate *f*.

A search neighborhood **N**_*k*_[*i* + 1] of *k*-th marker (the grey ball in Fig 1) for frame [*i* + 1] can be defined with respect to *k*-th marker position in frame [*i*], that is,

$$N_{k}\left[i+1\right]\equiv \left\{x|\|x-x_{k}\left[i\right]\|<r_{0}\left[i\right]\right\}.$$ (1)

Note that the radius of a search neighborhood *r*_0_[*i*] varies in time for each frame, which can be chosen with respect to the varying marker spacing. If distances between markers are relatively small, the search neighborhood should correspondingly small, but otherwise this requirement can be relaxed. It is obvious that the radius of a search neighborhood should have a proportional relation to the lower bound of marker spacings, therefore, introducing a proportional constant *K*, the search radius *r*_0_[*i*] can be represented with respect to the minimum marker spacing, that is,

$$r_{0}\left[i\right]=K\;{\text{min}}_{l,m}\;d_{l,m}\left[i\right].$$ (2)

### The minimal displacement condition and worst case scenario

In order to avoid marker confusion during a tracking process, the minimal displacement condition should be satisfied: either a marker moves slowly or the neighboring marker is sufficiently far away so that the marker cannot go beyond its search neighborhood within a short sampling interval. Then the following inequality defined for each frame [*i*] should hold for all markers:

$$\|x_{k}[i+1]-x_{k}[i]\|<r_{0}[i],\;\forall k.$$ (3)

Let us assume that at frame [*i*], our motion tracking is facing the worst case: the search radius could be chosen to have the minimum value due to the spacing of markers reaching their minimum, and one of the markers is travelling with the maximum speed during the whole motion tracking period. To satisfy the minimal displacement condition regardless of the worst sampling moment for the whole measurement period, the maximum travelling distance of any marker during a unit frame interval should not exceed its smallest search neighborhood selected for all frames. Then the following inequality with respect to the time index *i* should hold for all markers, that is

$$\max_{i}\{\|x_{k}[i+1]-x_{k}[i]\;\|\}<{\text{min}}_{i}\{r_{0}[i]\},\;\forall k.$$ (4)

Since the left side of the inequality is representing the global maximum of distance travelled during one frame step, it can be substituted with *v*_*max*_ ⋅ Δ*t*, where *v*_*max*_ is the global maximum speed for all markers for the whole measurement frames. Also taking the minimum search radius over all frames, we can get a “time-invariant” search radius *R*_0_, which denotes the strongest condition (smallest in distance), that is,

$$R_{0}={\text{min}}_{i}\left\{r_{0}\left[i\right]\right\}=Kd_{min}$$ (5)

where *d*_*min*_ is the global minimum spacing between all markers over all frames. The resulting inequality from Eq 4 is,

$$v_{max}\Delta t<Kd_{min}.$$ (6)

Finally, we obtain a sufficient frame rate criterion that can satisfy the minimal displacement condition,

$$f>\frac{1}{K}\frac{v_{max}}{d_{min}}.$$ (7)

### Tracking efficiency constant *K*

Non-dimensional constant *K* is defined as the ratio of the searchable travelling distance range of a marker to the minimum marker spacing in a unit time step *∆t*. We define this constant as “tracking efficiency constant”, which enables us to determine the performance or efficiency of the optical tracking system by means of distance-related concept. For example, if *K* is 0.5, it means that the motion tracking system is capable of tracking a marker travelling up to half distance of the minimum marker spacing in a frame interval. If the system has smaller *K*, the chance of losing the marker trajectory increases despite retaining the same spacing and speed because the possible search neighborhood is relatively small and the marker may go out of the region easily. This necessitates the optical tracking system to be driven with a higher frame rate in order to satisfy the Eq 7.

When the tracking efficiency constant is known (or given) for an optical motion tracking system, it is possible to predict the minimum frame rate that should be associated with a motion’s maximum speed and minimum spacing. For example, if *K* is 0.1, *v*_*max*_ is 1 m/s and *d*_*min*_ is 0.1 m, then the measurement requires a frame rate higher than 100 Hz.

## Experiments and Results

### Experimental Overview

**Fig 2:**
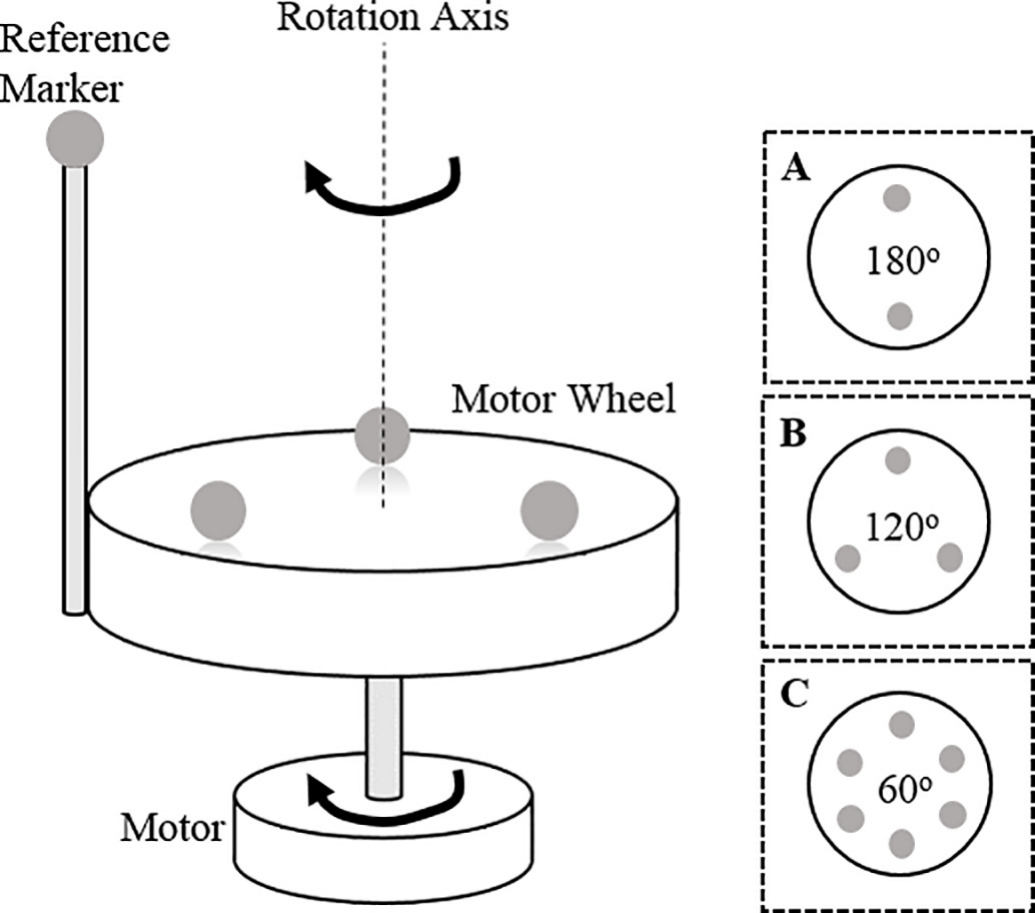
Optical marker placement for tracking the rotating motor. Multiple 7 mm light-weight passive markers were attached on the measurement plane (motor wheel) and rotating motions were tracked with 4 Qualisys 300 cameras. The reference marker on the rod was used to identify the reference marker R0 manually. Three marker spacing setups (A, B, C in the dotted squares) were used to give different marker spacing cases.

To verify the minimum frame rate proposed in the theoretical section, the rotating motion of 2.5W DC motor (Model: FT-758, Deltaco Ltd., Sweden) was captured with four Oqus300 motion tracking cameras (Qualisys Ltd., Gothenburg, Sweden). Attaching 7 mm passive markers on the flat surface of the motor’s wheel (Fig 2), conditions for successful motion tracking with respect to three factors–marker spacing, target motion’s speed, and device’s frame rate—were observed.

Throughout the experiments, three different marker spacing setups were used (A, B, C in Fig 2). Since the marker setups are symmetrical, a reference marker was additionally used for visual identification of markers, not for the internal tracking algorithm of Qualisys Tracking Manager (QTM) software. From the theoretical criterion of Eq 7, what our experiments aim to demonstrate is the inverse proportional relation between the minimum spacing of markers and the required frame rate for a successful motion tracking. Therefore the reference marker on the rod was positioned sufficiently far from the motor wheel in order to prevent overlapping with other markers on the measurement plane (Fig 2). For convenience, let us call the closest marker to the reference marker as R0 marker.

**Fig 3:**
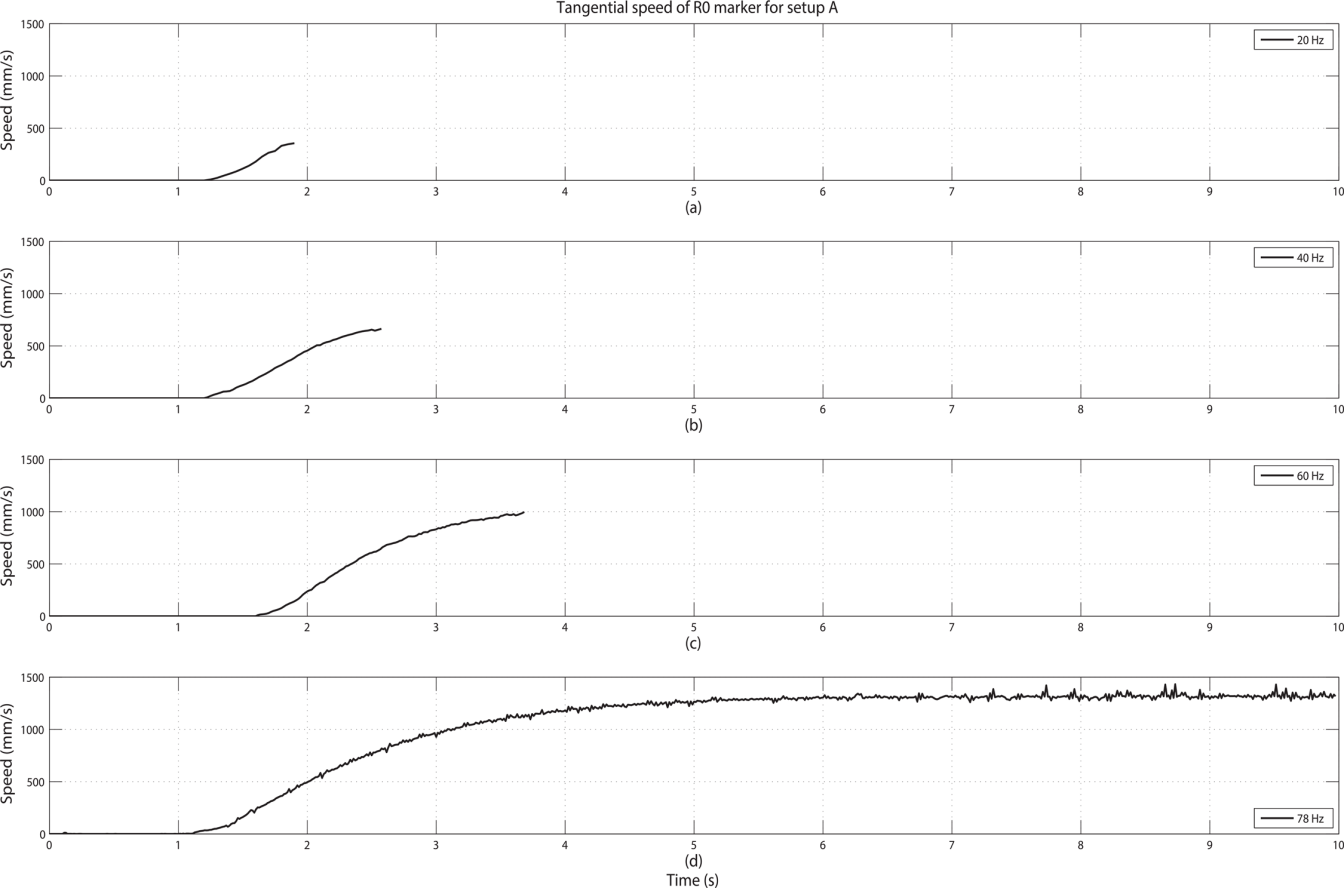
Tangential speeds of R0 marker for setup A with increasing frame rates. Frame rates were: (a) 20 Hz, (b) 40 Hz, (c) 60 Hz, (d) 78 Hz. Since 78 Hz is above the required frame rate for setup A, the full speed rotation was tracked throughout the whole 10 seconds recording in (d), but others showed termination during the recordings.

A rotating DC motor was chosen as the source of target motion because it can generate periodic motion with a direct relationship between motion frequencies and the rotating speeds. An example of tangential speed profile (from the start to full speed) of the motor used for the experiment can be found in Fig 3D. This speed curve was obtained from one of the successful tracking session for setup A with a 78 Hz frame rate for the motion tracking, plotting the trajectory of R0 marker which is attached about 20 mm from the rotational axis. The plotting shows that when the motor is turned on, the tangential speed of R0 starts from zero and reaches its full speed rotation within 6 seconds and then remains at the same speed level. Therefore, the duration of each session was selected as 10 seconds, which should cover the full speed rotating motion sufficiently. Due to the imperfections of the motor (2.5W±0.25W), the peak tangential speed of the motor had slight deviation varying from 1360 mm/s to 1440 mm/s for all the experiments. Note that even if we consider the fastest rotating of 1440 mm/s with 20 mm radius, the motor maximally rotates 9.81 times per second. Thinking of the NSSR, the target motion should be sufficiently captured with a frame rate around 20 Hz; however it would turn out to be insufficient for our multiple passive marker case.

Summarizing the overview of experiments: in Experiment 1, the minimum frame rates for the “successful tracking” were observed for each marker spacing by changing the frame rate of the optical motion tracking system. In Experiment 2, to validate Eq 7 with the maximum speed of a motion, frame rates of the motion tracking system were selected below the minimum frame rate (from Experiment 1) and intended the failure in recording before reaching the full speed rotation. Recalling the tangential speed curve of the motor in Fig 3D, the speed increases continuously in the transient interval before reaching the full speed rotation. When the frame rate is no longer sufficient to capture the increasing speed, the tracking of the rotating motion would fail and the speed at that moment was observed.

### Experiment 1: Frame rate and marker spacing relation

First, the minimum frame rate for the “successful tracking” of motion for three marker spacing cases (A, B, C in Fig 2) were observed. Note that a process of tracking markers in optical motion capture cannot be always 100% consistent even though we have good measurement conditions. A tracking ability can be limited if the calculation load is very high (high number of markers) compared to the computing power, but the Qualisys system used in this experiments can track up to 10,000 markers with a frame rate of 500 Hz. Therefore, we assume that the calculation load is negligible. Other than these factors, there are several internal factors that can affect the tracking process mostly due to the characteristics of tracking software such as an initial over-clocking (sampling) scheme of a motion tracking system (generally used for initial marker catch-up), and ghost marker appearance due to the position prediction error of a camera vision algorithm. Also there are some physical factors related to optical cameras (e.g. undesired IR reflections during measurement), or unexpected target motions such as unstable motor rpm in our case. To remove uncertainty from these experimental factors, the motor was turned on only after the over-clocking scheme of the motion tracking system had ended. Also, the motor wheel was coated with non-reflective materials so as to not cause any spurious reflections during the whole measurement. However, ghost marker appearance due to the internal algorithm error could not be controlled in the experiment because it is related with matrix inversion process of marker capturing and impossible to prevent for all cases. Also there remained some uncertainty of motor rotating speed varying for each session. Trying to eliminate these unwanted effects, we determined a recording as a “successful tracking” if the resulting marker trajectory output had a 100% perfect capture rate (without applying internal interpolation by the tracking software) throughout for five consecutive repeating sessions with a given frame rate. For each marker spacing setups, the minimum frame rate *f*_*N*_ was determined (down to a 1 Hz resolution) as the lowest frame rate that enabled the successful tracking. When the trackings were successful for five consecutive sessions, the maximum instantaneous tangential speed for five sessions were averaged and defined as *v*_*max*_.

**Fig 4:**
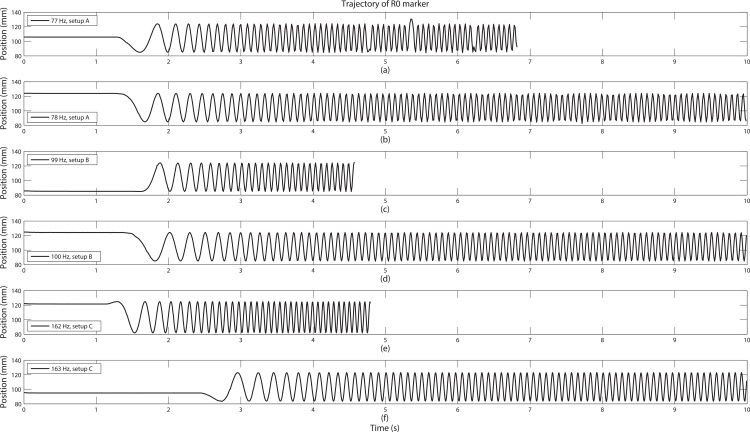
Comparison of trajectories for the successful tracking sessions and failing sessions. From the top, the measurement conditions were (a) 77 Hz, setup A, (b) 78 Hz, setup A, (c) 99 Hz, setup B, (d) 100 Hz, setup B, (e) 162 Hz, setup C, and (f) 163 Hz, setup C. For the frame rates below the selected *f*_*N*_ values, trajectories were truncated before reaching the full speed rotation.

The observed minimum frame rate *f*_*N*_ was 78 Hz, 100 Hz, and 163 Hz for corresponding setups A, B, and C (see Table 1) and we can see that marker trajectories of R0 were tracked 100% for 10 seconds (Fig 4B, 4D and 4F). But for sessions with frame rates just 1 Hz below to the obtained minimum frame rates, failure of tracking occurred (Fig 4A, 4C and 4E).

**Table 1:** Observation results for Experiment 1 and Experiment 2.

Marker Setup	Successful sessions (Exp. 1)			Failing sessions (Exp. 2)				
	*f*_*N*_ (Hz)	*v*_*max*_ (mm/s)	*d*_*min*_ (mm)	*f*_*S*_ (Hz)	*v*_*fail*_ (mm/s)	*d*_*min*_ (mm)	Slope	*K*
A(180˚)	78	1363.29	38.93	20	336.99	38.83	16.54	0.43
				40	656.83			
				60	993.20			
B(120˚)	100	1408.39	35.45	20	293.04	36.67	14.18	0.39
				40	603.96			
				60	847.11			
				80	1116.73			
C(60˚)	163	1387.58	20.77	20	174.02	23.34	9.42	0.40
				40	400.64			
				60	617.89			
				80	808.03			
				100	957.15			
				120	1178.18			
				140	1286.08			
				160	1439.77			

For Experiment 1, *f*_*N*_ is the minimum observed frame rate, *v*_*max*_ is the maximum tangential speed, and *d*_*min*_ is the minimum marker spacing used. For Experiment 2, *f*_*S*_ is the selected frame rate for the session and *v*_*fail*_ is the failing speed. Slope values were calculated with least square approximation method. The tracking efficiency constant *K* for each setup was estimated from *d*_*min*_ and the slope value.

For the successful sessions, the tangential speed deviations were quite small (within 5%) for varying setups, but more than double frame rate was needed for the closest spacing (marker setup C) compared to the largest spacing (marker setup A) (see Table 1). This implies that although we may have cases of motion with the same speed, a sufficient frame rate for a successful motion tracking should be selected with respect to the passive markers’ spacing. It should be noted that these observed frame rates are significantly above the NSSR. Since the motor motion has the maximum frequency component at less than 10 Hz, one could argue that the double of the maximum frequency should be enough to track the motion, but what our work has revealed is that we need far higher frame rates (78 Hz to 163 Hz) than the NSSR for successful measurements when using passive markers.

**Fig 5:**
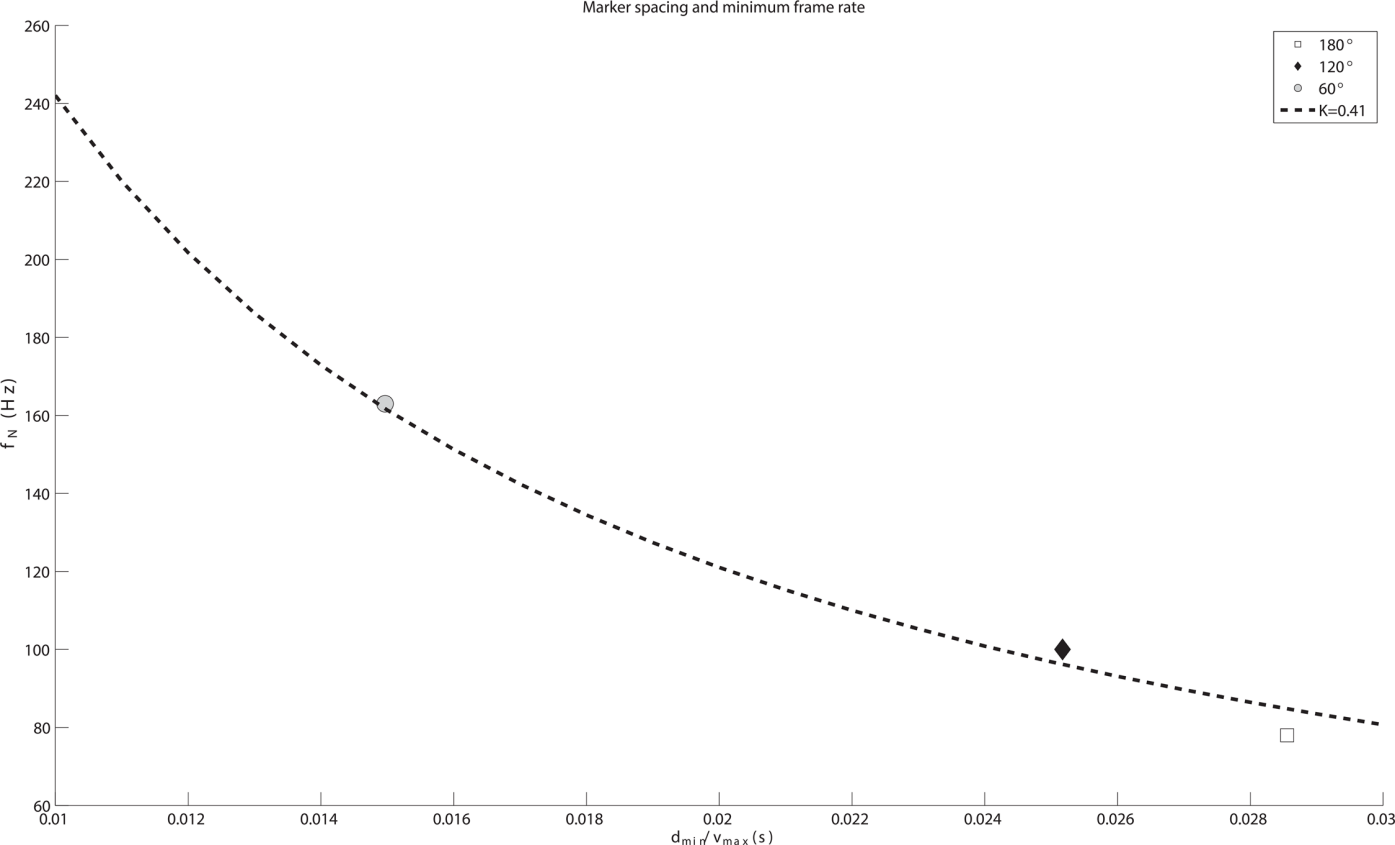
Relation of the minimum frame rate with minimum marker spacing. A paired data (*d*_*min*_/*v*_*max*_, *f*_*N*_) is plotted for each marker spacing case and fitted to the inverse proportional curve: *y* = 1 / *Kx*. The dotted curve shows the least squared fit curve and *K* is 0.41. The region above the curve means the successful tracking region (*f*_*N*_ > *v*_*max*_/*Kd*_*min*_) and region below denotes the tracking failure.

Fig 5 shows a relation of *f*_*N*_ to the minimum marker spacing normalized via maximum speed (*d*_*min*_/*v*_*max*_), which has the dimension of time. The physical translation of *d*_*min*_/*v*_*max*_ on the *x*-axis is the time duration needed for a marker to travel the minimum marker spacing with the maximum speed. In Fig 5, there are three data points (*d*_*min*_/*v*_*max*_, *f*_*N*_) from Experiment 1 (see Successful sessions in Table 1), clearly following the inverse proportional curve. The dotted curve in Fig 5 shows the least square curve fit to satisfy the inverse proportional relation (*y* = 1 / *Kx*). The result shows that our Qualisys motion tracking system has the tracking efficiency constant *K* of 0.41, capable of tracking a fast moving marker that travels up to 41% of the minimum spacing in a unit frame interval.

### Experiment 2: Frame rate and speed relation

To verify the frame rate and speed relation in Eq 7, the system’s frame rate *f*_*S*_ was changed manually, starting from 20 Hz and increased with an increment of 20 Hz until *f*_*S*_ reaches the minimum frame rate *f*_*N*_ observed in Experiment 1. At the failing moment of tracking, marker R0’s tangential speed was recorded. Three measurements for setup A (*f*_*S*_: 20,40,60 Hz), four for setup B (*f*_*S*_: 20,40,…,80 Hz), and eight for setup C (*f*_*S*_: 20,40,…,160 Hz), in total 15 sessions, were measured (Table 1). Because this experiment was performed after Experiment 1 was completed, markers had some position differences from the previous setup on the motor wheel. But since the marker’s position can always be accurately captured with the motion tracking system, results of Experiment 2 were calculated with new positions as shown in Table 1.

The three upper plots (Fig 3A–3C) show the typical trend of tangential speed curves of R0 marker from the starting point to the failing moment for setup A. We can see that for increasing frame rates, the system can handle higher speeds. The speed examples in Fig 3 only show the results of marker setup A, but this trend holds for all spacing setups (see Failing sessions in Table 1). When the marker spacing is increased, tracking can successfully capture faster motions.

**Fig 6:**
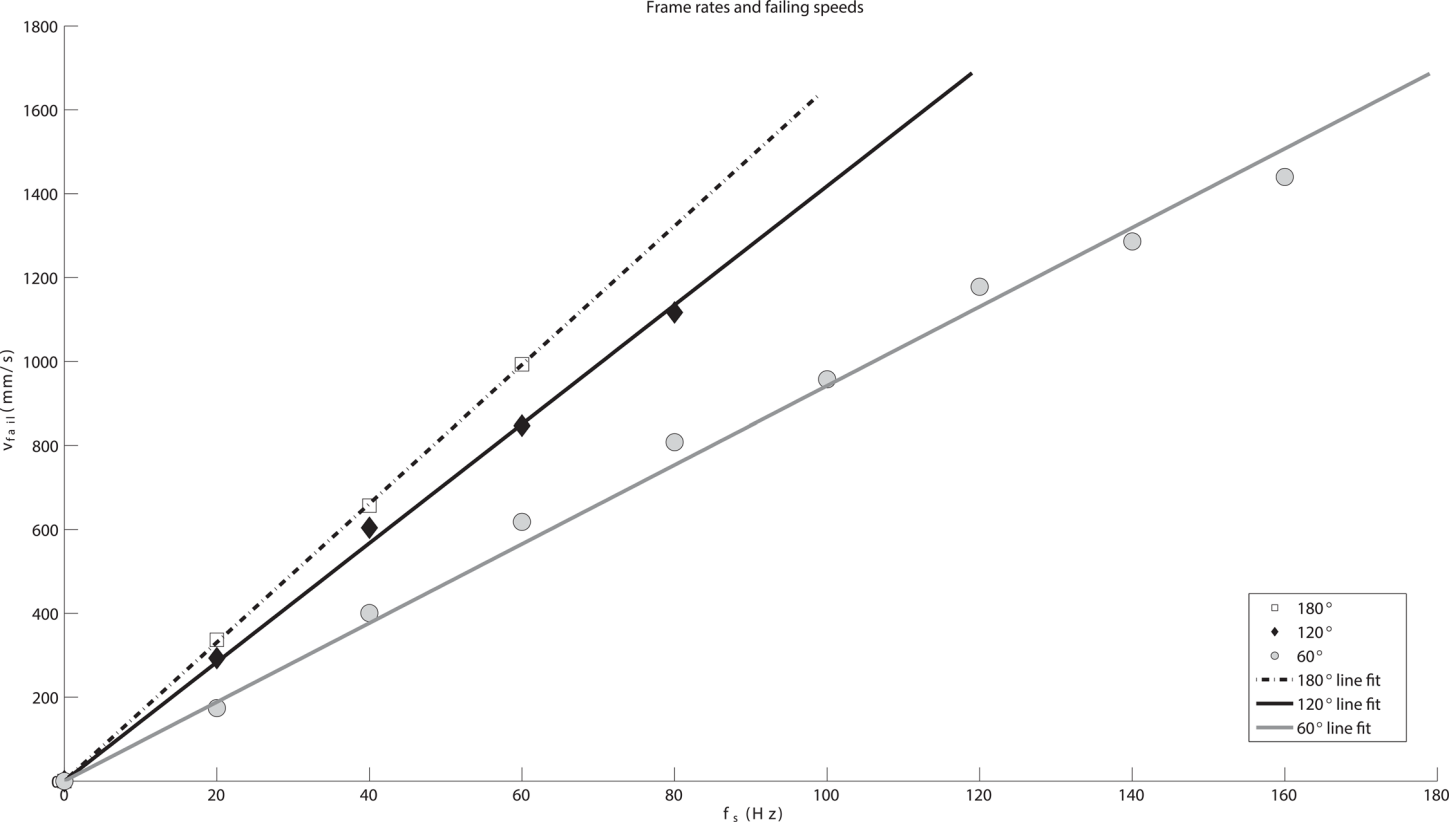
Relation of the minimum frame rate and the maximum speed of markers. Paired data set of frame rate to failing speed (*f*_*S*_, *v*_*fail*_) is plotted for each spacing case and fitted with linear least square curve. Lines show the least squared fit for three marker spacings and the ratio between slopes are close to the minimum marker spacings.

Fig 6 summarizes Experiment 2 for all marker setups, and it is clear that the tracking failing speed *v*_*fail*_ is proportional to the device’s frame rate regardless of marker spacing setup. Calculating the linear fitting lines for the observed data using the least square method, the slopes were 16.54 for setup A, 14.18 for setup B, and 9.42 for setup C. Note that these slopes (*K* ⋅ *d*_*min*_) have the length dimension (unit in mm). A line with higher slope value means that the tracking can hold up to higher speeds although the device is running with the same frame rate. For example, when *f*_*S*_ is 60 Hz, we can see that marker setup A, with the minimum marker spacing of 38.33 mm, can hold up to 993.20 mm/s, while setup C, with 23.34 mm spacing, is only bearable up to 617.89 mm/s. The ratio between the three slopes is very close to the actual *d*_*min*_ ratio (Table 1) which implies that the tracking efficiency remain quite constant for different sessions. Dividing the slope value with the minimum spacing for each setup, the average of *K* is 0.41, which perfectly matches with the least squared curve result from Experiment 1 that is shown in Fig 5.

## Discussion

From the two experiments, it is clear that our theoretical derivation of the minimum frame rate criterion in Eq 7 holds true. The rate should count on the spacing between the markers. It gives an interesting finding that when using passive markers for optical motion tracking, our famous guideline for determining the minimum frame rate—Nyquist-Shannon sampling rate—is insufficient for the passive marker case because the spacing between markers is not considered. Determining a suitable frame rate–which may be the most important element to determine before taking any kind of measurement in signal processing contexts–requires an observer to know in advance how fast the target object is moving. For the motion tracking, this means having some preliminary estimation of the maximal speed of a target object. But in the case of using passive markers, this estimation of “fastness” is also closely related to the spacing between markers and the abovementioned inverse proportional relation holds. Although a body moves at the same speed, if the marker spacings are small, the motion is relatively fast and requires higher frame rates in order to be tracked successfully. Therefore, the “fastness” of a passive marker's motion is a relative phenomenon.

The spacing condition of just a few centimeters (20 mm to 40 mm) used in the experiments reported above may be considered too harsh for practical human motion tracking purposes. However, for the purpose of measuring detailed human motion, such as finger motion or facial motion, these marker spacings are clearly within the necessary range. In such kinds of motion tracking cases, the frame rate should be selected accordingly because the inverse proportional relation of marker spacing in Eq 7 can make the rate exceed the camera’s sampling capability. Recall that the 10 Hz motor motions used in our experiments can be considered very slow compared to the sampling capability of high-quality optical motion tracking systems that is around 500 Hz nowadays. However, the 10 Hz motor turned out to require a frame rate of more than 160 Hz in some cases. For faster moving and closely spaced markers, even a couple of hundred hertz may in some cases not be sufficient.

The tracking efficiency constant *K* proposed in this paper may be considered a general performance measure for optical motion tracking devices. Initially, the performance of an optical tracking system can be assessed by given hardware specification such as the cameras' view angle, image sensor resolution, measurement distance range, or frame rate range, etc. However, the tracking efficiency constant we have presented here demonstrates a possibility to evaluate the internal tracking algorithm performance quantitatively, something which can be understood intuitively with two simple distance features: the search radius and the minimum marker spacing.

## Supporting Information

S1 Dataset Raw dataset for Fig 3. Velocity data used in Fig 3 are extracted from Qualisys Track Manager (QTM) software with MAT file format (Total 4 files included).
(ZIP)

S2 Dataset Raw dataset for Fig 4. Trajectory data used in Fig 4 are extracted from Qualisys Track Manager (QTM) software with TSV file format with standard mocap data structure (Total 6 files included).
(ZIP)

S3 Dataset Raw dataset for Fig 5. Velocity data used in Fig 5 are extracted from Qualisys Track Manager (QTM) software with MAT file format (Total 15 files included).
(ZIP)

S4 Dataset Raw dataset for Fig 6. Velocity data used in Fig 6 are extracted from Qualisys Track Manager (QTM) software with MAT file format (Total 15 files included).
(ZIP)
